# Relevance of Glucagon-Like Peptide 1 (GLP-1) in Inflammatory Bowel Diseases: A Narrative Review

**DOI:** 10.3390/cimb47050383

**Published:** 2025-05-21

**Authors:** Antonietta Gerarda Gravina, Raffaele Pellegrino, Michele Izzo, Ilaria De Costanzo, Giuseppe Imperio, Fabio Landa, Assunta Tambaro, Alessandro Federico

**Affiliations:** Hepatogastroenterology Division, Department of Precision Medicine, University of Campania Luigi Vanvitelli, Via L. de Crecchio, 80138 Naples, Italy

**Keywords:** GLP-1, inflammatory bowel disease, type 2 diabetes mellitus, obesity, diabetes, gut inflammation, epithelial barrier, gut microbiota

## Abstract

Inflammatory bowel diseases (IBDs) are complex immune-mediated disorders characterised by an unpredictable direction and commonly associated metabolic comorbidities along with obesity and type 2 diabetes mellitus (T2DM). Recent evidence has highlighted the therapeutic capacity of glucagon-like peptide 1 receptor agonists (GLP-1 RAs), already employed in treating T2DM and obesity, in modulating systemic and intestinal inflammatory responses. This narrative review examines the general organic traits of GLP-1, with a specific awareness of its primary gastrointestinal actions and the efficacy of GLP-1 RAs in promoting weight loss and dealing with glycaemic control, mainly in sufferers with IBD. Furthermore, the effects of those agonists on the progression of IBD, their protection profile, their impact on bowel preparation for endoscopic procedures, and their therapeutic capacity, supported through preclinical and early clinical studies, are discussed. GLP-1 RAs appear to lessen the intestinal inflammatory burden by enhancing intestinal epithelial barrier features and modulating the gut microbiota. However, further clinical research will be necessary to verify whether GLP-1 RAs could play a position in IBD treatment.

## 1. Introduction

Inflammatory bowel diseases (IBDs) are chronic, immune-mediated conditions that lead to both short- and long-term complications (including cancer) due to sustained immune activation over time, with a course that is not always predictable and often characterised by remissions and relapses [1].

The pathogenesis is highly complex, involving impairments of the immune system—particularly the T-helper 1 and T-helper 17 pathways—alterations in the gut microbiota and other emerging metabolic-related regulatory systems such as the melanocortin system [1,2]. The Janus Kinase-Signal Transducer and Activator of Transcription pathway is another extensively studied pathway for which pharmacological modulators have been developed in IBD’s pathogenesis [3].

The current therapeutic armamentarium for IBD is vast and includes conventional therapies based on gut-targeting anti-inflammatories, immunomodulators, and advanced therapies (biologics and small molecules); where medical treatment is not feasible, surgical intervention remains an option [4,5,6,7,8]. Nevertheless, a significant proportion of patients—up to and beyond 40%—fail to achieve the predefined therapeutic outcomes, thereby establishing a complex therapeutic ceiling that is difficult to overcome [9].

This necessitates the continuous search for new therapeutic targets to identify more profound and effective mechanisms of action. Moreover, in addition to the multiple and systemic extra-intestinal manifestations to which they may be exposed, patients with IBD frequently present with metabolic comorbidities such as type 2 diabetes mellitus (T2DM) or obesity. The latter has been shown to worsen the course of IBD, increasing the risk of hospitalisations and infectious events and, not least, raising the healthcare costs associated with patient management [10].

Moreover, extensive population-based studies, such as that by Jess et al. [11] conducted on over six million Danes, identified a standardised incidence ratio (SIR) of T2DM in patients with IBD of 1.54 (95% CI 1.49–1.60), demonstrating over one thousand more cases than expected in the IBD cohort, particularly among patients with ulcerative colitis (UC) compared to those with Crohn’s disease (CD), with the highest risk observed within the first year after IBD diagnosis (SIR 4.48). This increased risk of T2DM has also been confirmed by a subsequent meta-analysis [12]. In addition, obesity is also a highly prevalent comorbidity in patients with IBD [13], and associated conditions such as non-alcoholic fatty liver disease [14] are likewise common. All these conditions, however, are far from being distinct clinicopathological entities; instead, they converge within the complex framework of metabolic syndrome, which, according to available meta-analytic data, has a pooled prevalence of 19.4% (95% CI 15.1–23.8%) in patients with IBD, with a greater epidemiological burden among patients with UC compared to those with CD (38.2% vs. 13.6%) [15]. All of this also entails a non-negligible risk of progression to associated cardiovascular complications [16].

To date, the metabolic therapy of T2DM is expanding beyond classic oral hypoglycaemic agents, such as insulin sensitisers and secretagogues, by incorporating modulation of the incretin system through glucagon-like peptide-1 (GLP-1) receptor agonists (GLP-1 RAs) [17].

In light of all this, the aim of this narrative review is, starting from the structural and functional principles of GLP-1, to examine the effectiveness of the pharmacological modulation of GLP-1 through specific agonists, both for the management of weight loss, specifically in patients with IBD, and to assess the available evidence on the impact of such treatment on disease activity and its safety in patients with IBD, including the influence of various relevant parameters such as bowel preparation before endoscopic procedures. Finally, this review will focus on the specific therapeutic potential emerging from preclinical and direct clinical data of these pharmacological agents in IBD.

## 2. GLP-1: General Characteristics and Production

### 2.1. Principles of GLP-1 Structure and Primary Metabolic Effects

GLP-1 is tandemly linked to GLP-2 within a gene discovered in the early 1980s, which encodes a larger peptide, proglucagon [18]. GLP-1 is continuously produced by enteroendocrine cells in response to food intake, at which point, in the postprandial phase, its levels increase dramatically compared to preprandial and generally interprandial levels [18].

**Figure 1:**
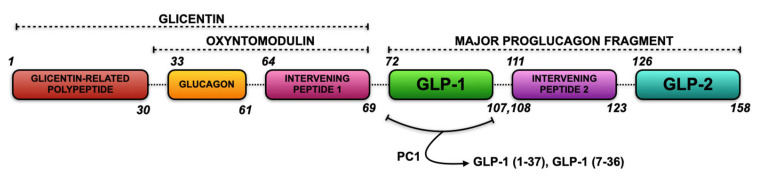
Structure of human proglucagon’s complementary DNA (cDNA) and indication of the enzymatic production of glucagon-like peptide 1 (GLP-1). Human proglucagon comprises several fragments identifiable on its cDNA, and combinations give rise to various peptide fractions. The glicentin-related polypeptide, glucagon, and intervening peptide 1 constitute glicentin. In the absence of the glicentin-related polypeptide, the latter gives rise to oxyntomodulin. In contrast, the combination of GLP-1, intervening peptide 2, and GLP-2 constitutes the major proglucagon fragment. Intestinal prohormone convertase 1 (PC1) cleaves this large fragment, yielding only the two bioactive forms of GLP-1, namely, GLP-1 (7-37) and GLP-1 (7-36) amide.

From the post-translational processing of proglucagon, the two bioactive forms of GLP-1 are obtained, namely, GLP-1(7-37) and GLP-1(7-36), the latter being the predominant form (Figure 1) [19]. These are produced by the action of prohormone convertase (PC1/3) in the intestinal tract, owing to the L cells of the small intestine and adipose tissue [19]. Moreover, GLP-1 has a very short half-life in circulation (usually up to approximately two minutes) because it is rapidly inactivated by the enzyme dipeptidyl peptidase-4 (DPP-4) [19].

GLP-1 is involved in the regulation of multiple metabolic biological functions. It controls glucose-dependent insulin secretion at the pancreatic endocrine islets level while inhibiting glucagon secretion [20]. These insulinotropic functions have prompted the development of multiple GLP-1R agonists for treating type 2 diabetes mellitus [21]. Interestingly, GLP-1R agonism does not directly affect insulin signalling and glucose uptake in the primary responsible organs (muscles, liver, and adipose tissue). Therefore, it has been hypothesised that GLP-1R activation at the peripheral nerves and blood vessels level may enhance tissue blood flow, thereby indirectly regulating insulin function by increasing insulin distribution and recruitment within the muscle microcirculation [22].

The other primary metabolic function of GLP-1 is the regulation of hunger, resulting in reduced food intake by patients and consequent weight loss. While in murine models, GLP-1R agonism has been shown to activate brown adipose tissue, increasing sympathetic energy expenditure and reducing peripheral adipose deposits [18], in humans, a complex neural interaction involving the central nervous system has also been demonstrated [23]. Specifically, the activation of GLP-1 functions in various brain regions triggers different responses that promote these effects. Areas associated with reduced hunger appear to include the arcuate nucleus and the paraventricular nucleus of the hypothalamus, with the lateral hypothalamic area also contributing, particularly in lowering short-term hunger [23]. Conversely, the sensation of pleasure associated with the desire for palatable food is diminished through the involvement of the mesolimbic reward circuit. At the same time, the hippocampus is implicated in reducing the intake of high-fat foods [23]. Finally, satiety is also induced by decreasing the motivation to seek food through the involvement of the hindbrain [23].

### 2.2. Gastrointestinal Regulation Induced by GLP-1

GLP-1, through its interaction with its receptors, can modulate various actions affecting the gastrointestinal tract. Once produced by enteroendocrine cells of the gut, it can contribute to anti-hunger actions by modifying gastrointestinal motility and slowing gastric emptying [24]. However, this action does not affect overall gastric emptying but primarily occurs within the first postprandial hour.

Specifically, duodenal enteroendocrine cells, in response to food intake, release GLP-1 and cholecystokinin (CCK), which, in addition to entering the circulation to exert extragastrointestinal functions, promote the activation of vagal afferents where GLP-1R are located [25,26]. It is known that the modulation of motility by GLP-1 occurs by impairing gastric accommodation (as observed when subcutaneous administration of the analogue liraglutide is performed [27]). On the other hand, GLP-1 activates nitrergic nerve afferents that slow gastric emptying, as demonstrated by Andrews et al. [28], who observed that following GLP-1 administration in healthy volunteers, fasting gastric volumes increased along with an enhancement in postprandial accommodation. This occurs through a reduction in peristaltic waves and the muscle tone of the gastric fundus [29,30].

However, the regulatory action of GLP-1 is not limited to the gastric fundus; it is also known that fasting antral contractility episodes can be abolished entirely for approximately four hours following parenteral infusion of GLP-1 in healthy volunteers [31].

More distally, it has been observed that GLP-1 can reduce the number of migrating motor complexes at the jejunal level in healthy subjects by decreasing the amplitude, frequency, and duration of intestinal motor contractions [31,32].

However, it should not be overlooked that an even more distal role of GLP-1 has also been hypothesised as a regulator of motility alongside peptide YY through a concept postulated as the “ileal brake” [33]. This inhibitory brake is induced by nutrients in the terminal ileum, which exerts a braking action on ileocolic motility to facilitate nutrient absorption [33]. This mechanism is hypothetically attributable to vagal phenomena through the activation of GLP-1R located at the ileocolic level [33,34].

## 3. Effectiveness of GLP-1 RAs for T2DM Treatment and Weight Loss in Patients with IBD

As mentioned above, the use of GLP-1 RAs is currently approved by both the European Medicines Agency (EMA) and the Food and Drug Administration (FDA) for use in T2DM and obesity [35,36]. Overweight, obesity and T2DM are rapidly increasing worldwide and represent a growing public health challenge as they severely impact quality of life and increase the risk of cardiovascular disease, hypertension, dyslipidaemia, and other chronic complications [37]. According to current estimates, the global prevalence of T2DM was 5.9% in 2021 and is expected to rise to approximately 9.5% by 2050, corresponding to 1.27 billion people with T2DM in that year [38]. Although IBD often leads to weight loss, malnutrition, and sarcopenia, the prevalence of obesity in this group aligns with the overall rates observed in the general population [39]. Indeed, despite many patients being found to be underweight at the time of diagnosis of CD or UC, 15–40% of adult patients with IBD are obese (body mass index, BMI ≥ 30 kg/m^2^), and an additional 20–40% are overweight (BMI 25–29.9 kg/m^2^) [39,40]. In detail, visceral obesity rather than BMI appears to negatively affect treatment response and increase the risk of recurrence and surgical complications [41,42,43].

Over time, multiple GLP-1 RAs have been developed, including examples such as tirzepatide, mazdutide, orforglipron, semaglutide, retatrutide, dulaglutide, litaglutide, albiglutide, exenatide, and lixisenatide. The broad availability of these agents, some of which are also administered orally (e.g., semaglutide), has created the need to compare them concerning various relevant metabolic outcomes, such as glycaemic control, body weight management, and lipid profile. Yao et al. [44] conducted a meta-analysis concluding that tirzepatide was the most effective in controlling glycaemia (with a mean difference in haemoglobin A_1c_ of −2.10%). At the same time, CagriSema (i.e., semaglutide with cagrilintide) was most effective in reducing body weight (with a mean difference of −14.03 kg).

The effectiveness of GLP-1 RAs in treating T2DM and obesity has been well documented in large clinical trials in the general population [45,46,47]. Although studies demonstrating efficacy and safety in the IBD subpopulation are limited, available data suggest that this class of drugs maintains therapeutic effectiveness in the treatment of T2DM and weight loss in patients with UC or CD.

In a retrospective cohort study conducted by Desai et al. [48], the effectiveness and safety of semaglutide in reducing body weight in obese patients with IBD was evaluated in comparison to a cohort of non-IBD patients not suffering from other chronic inflammatory diseases. The primary endpoint was the change in mean total body weight (TBW) between 6 and 15 months after initiation of semaglutide therapy compared with baseline between the two cohorts of patients. The change in mean TBW after initiation of semaglutide in the IBD cohort was −16 ± 13.4 pounds versus −18 ± 12.7 pounds in the non-IBD cohort (*p* = 0.24). Furthermore, a subgroup analysis was also performed according to IBD type (i.e., CD and UC) and IBD medication considering patients on advanced drug therapy or 5-aminosalicylic acid derivatives (5-ASA), showing that semaglutide use resulted in similar mean TBW loss in the two cohorts, with a mean percentage of TBW after semaglutide that was about −7% between 6 and 12 months and −9% between 12 and 15 months. In addition, the subgroup analysis showed no difference in the change in mean TBW observed in the UC and CD cohort compared to the non-IBD cohort, and no difference in the change in mean TBW was observed in the IBD cohort treated with advanced therapies and the IBD cohort treated with 5-ASA compared to the non-IBD cohort.

The retrospective study by Ramos Belinchón et al. [49] evaluated the effectiveness of semaglutide and liraglutide in reducing TBW after 6 months of treatment. The study collected data from 16 obese patients with IBD (nine patients diagnosed with CD and seven with UC) who were started on liraglutide 3 mg or semaglutide 1 mg therapy. After six months of therapy, 58.3% of patients had achieved a 5% or greater reduction in total body weight, with a median percentage loss of −6.2% (*p* = 0.002). In contrast, no statistically significant differences were found in laboratory parameters, which were evaluated as a secondary outcome.

Lastly, another single-centre observational study [50] recruited 36 non-diabetic, obese patients with IBD (i.e., 24 with CD and 12 with UC) on GLP-1-based therapy for weight control. Most patients (i.e., 32 out of 36) received semaglutide, while four patients were started on tirzepatide, which has a dual agonism function on GLP-1 and glucose-dependent insulinotropic polypeptide (GIP) receptors [51]. This study showed an 11.5% reduction in total body weight (*p* = 0.01) from baseline to the most recent weight available and a decrease in BMI from 34.0 kg/m^2^ to 31.0 kg/m^2^ (*p* < 0.001) [50].

In conclusion, all the data emerging from the mentioned studies show the effectiveness of GLP-1 RAs in obese and overweight patients, confirming that no substantial differences are observed in their use in patients diagnosed with IBD. In contrast, no statistically significant results were observed regarding blood glucose level control.

## 4. Impact of GLP-1 RAs on the Disease Course and Safety in IBD

GLP-1 RAs appear to play a protective role in systemic inflammation, both indirectly through improved glucose regulation and body weight loss and directly by binding to GLP-1 receptors on peripheral immune cells [52], thus reducing circulating levels of C-reactive protein (CRP) and tumour necrosis factor α (TNF-α) [53,54]. Studies on animals have shown that GLP-1 and its analogues reduce inflammation in the gut and the gastrointestinal tract with a consequent lowering of the expression of proinflammatory cytokines (e.g., TNF-α, interleukin (IL)-1α, IL-1β, and macrophage colony-stimulating factor) in chemically induced rodent models of colitis [55,56]. In addition, it has been demonstrated that in healthy humans, the density of GLP-1-producing L cells is highest in the distal ileum and rectum [57], which represent the disease sites most frequently involved in CD and UC, respectively, thus making GLP-1 RAs potential allies in IBD management.

The current scientific literature indicates that GLP-1 RAs are protective in reducing disease activity severity, hospitalisations, and IBD-related surgery [58,59,60]. Based on the US Collaborative Network in the TriNetX platform, Desai et al. [58] conducted a retrospective study in which data were collected from 5745 patients with UC and 5884 with CD. All patients had T2DM and were taking hypoglycaemic therapy. In detail, 1130 patients in the UC group and 1140 in the CD group were taking GLP-1 RAs, while the others were administered with other types of diabetes treatments [i.e., sulfonylureas, DPP-4 inhibitors, sodium-glucose co-transporter 2 inhibitors (SGLT2i), thiazolidinediones or metformin]. The study aimed to evaluate the use of intravenous steroids and IBD-related surgery, both as single and as composite outcomes. In the UC cohort, the results showed a lower risk of undergoing colectomy in the GLP-1 RAs group than in the control group [adjusted hazard ratio (aHR): 0.37, 95%CI 0.14–0.97], while there was no difference in the risk of the composite outcome (aHR: 1.12, 95%CI 0.86–1.45) or in the risk of intravenous steroid use (aHR: 1.21, 95%CI 0.92–1.59). The same protective effect on IBD-related surgery was observed in the CD cohort when compared to the control group [adjusted odds ratio (aOR): 0.55, 95%CI 0.36–0.84], but also, in this case, no difference was observed in the composite outcome (aHR: 0.97, 95%CI 0.78–1.22) or in the risk of intravenous steroid use (aOR: 1.04, 95%CI 0.80–1.34).

In another retrospective study conducted on the Danish population by Villumsen et al. [59], a lower risk in the composite outcome of the need for oral corticosteroids, TNF-α inhibitors, IBD-related hospitalisation, and IBD-related surgery was found [adjusted incidence rate ratio (IRR): 0.52, 95%CI 0.42–0.65]. Furthermore, the analysis of single outcomes highlighted that the need for oral corticosteroids and hospitalisation was lower among patients who had been treated with GLP-1 RAs and/or DPP-4 inhibitors compared to other antidiabetic drugs, with adjusted IRRs of 0.54 (95%CI 0.41–0.70) and 0.73 (95%CI 0.58–0.91), respectively. Although not statistically significant, a lower risk of surgery and a lower likelihood of starting anti-TNFα biologic therapy were also highlighted.

On the other hand, Levine et al. [60] found no statistical differences in the rates of IBD exacerbation (i.e., IBD-related hospitalisation, corticosteroid prescription, medication escalation or changes, or surgery) when comparing the 12 months before and the 12 months post-GLP-1 RAs initiation in a cohort of 224 IBD patients.

### What Is the Safety Profile for GLP-1 RAs?

Notwithstanding the potential benefits, inherent limitations must be considered when pharmacologically employing GLP-1 RAs [61].

Despite their potential benefits to the disease course, careful attention should be given when GLP-1 RAs are used in IBD patients since their most common side effects involve the gastrointestinal tract, with nausea, vomiting, diarrhoea, and constipation being the most frequently reported [62,63]. In particular, in terms of frequency, diarrhoea and nausea are the most common adverse events (frequency ≥ 1/10), followed by vomiting, constipation, dyspepsia, and abdominal pain, which are also relatively frequent (estimated frequency between ≥1/100 and <1/10) [61]. Very common, particularly with GLP-1 RAs administered subcutaneously, are skin reactions such as rash, erythema, or pruritus at the injection site. However, in most cases, these reactions are self-limiting and do not require specific treatment or discontinuation of the GLP-1 RA used [61].

Another extensively studied aspect is pancreatic injury, as some studies have shown that patients with T2DM taking GLP-1 RAs exhibited a higher incidence of hyperamylasaemia or hyperlipasaemia than controls [64]. However, a potential bias exists, as a subsequent analysis of the LEADER trial highlighted that, at baseline, among nearly ten thousand enrolled patients, approximately 17% already had elevated lipase levels and nearly 12% elevated amylase levels [65]. This makes these parameters less indicative of iatrogenic pancreatic insult unless the criteria of values tripled above the upper limit are met, which are necessary to support a diagnosis of acute pancreatitis. Further complicating the picture in patients with IBD is the possibility of asymptomatic hyperamylasaemia, which may occur independently [66,67]. Moreover, an increase in pancreatic enzymes is also associated with drugs commonly used to treat IBD, such as 5-aminosalicylic acid derivatives and conventional immunosuppressants [68,69]. Finally, a patient who already has an underlying metabolic disorder, such as T2DM or dyslipidaemia, inherently carries a higher risk of developing pancreatitis [70,71].

Moreover, although GLP-1 RAs have been associated with a modest increase in heart rate, the frequency of cardiovascular events compared to controls is, at present and based on available data, comparable [61]. An additional element of relevance when treating patients with IBD is the infectious risk associated with GLP-1 RAs, as these patients often undergo concomitant advanced treatment with biological agents and small molecules, which carry a specific infectious risk that must be considered [72]. Indeed, the registration trials of GLP-1 RAs have reported the possibility of upper respiratory tract infections and urinary tract infections, although no definitive cause–effect relationships have been established, particularly concerning clinically significant or serious infections [61].

Even though adverse events and their frequency in IBD patients are reported to be similar to the general population [73] and in the study by Desai et al. [48], no differences emerged in the risk of oral steroid use (aOR: 0.81; 95%CI 0.48–1.36), intravenous steroid use (aOR: 0.69; 95%CI 0.29–1.61) or advanced therapy initiation (aOR: 1.03; 95%CI 0.41–2.60) between IBD patients treated with semaglutide and controls.

Furthermore, conflicting data have emerged about the risk of intestinal obstruction in the general population [74,75,76], while in an extensive nationwide Danish study conducted explicitly on the IBD population, no increased risk of ileus or intestinal obstruction was seen in UC and CD (UC: aHR 0.42; 95%CI 0.21–0.86; CD: aHR 0.74, 95%CI 0.42–1.30) [77].

## 5. What Is the Role and Impact of GLP-1 RAs on the Optical Parameters Associated with Endoscopic Examinations and Adequate Bowel Preparation?

Patients with IBD frequently undergo endoscopic techniques for various purposes, ranging from the initial diagnosis to the monitoring of disease activity over time and the response to established therapies, as well as for the surveillance of IBD-associated cancer and for carrying out both diagnostic and operative procedures [78,79,80].

In addition to the potential impact on disease course, a secondary issue that must be considered in IBD management is the influence of GLP-1 RAs on bowel preparation for colonoscopy and video capsule endoscopy (VCE) given the slower gastric and intestinal transit observed in patients on GLP-1-based therapy [81]. Additionally, it is also well known that these patients often require particular attention, primarily due to disease-related endoscopic activity, both in terms of the type of bowel preparation to be recommended—to avoid mucosal damage caused by higher osmolarity at the expense of lower volume—and in light of the increased risk of suboptimal bowel preparation, which is especially detrimental in the surveillance of colitis-associated cancer [82,83,84]. A retrospective study [85] aimed to evaluate the quality of bowel preparation in patients taking GLP-1 RAs. Six-thousand two-hundred thirty-five patients were selected and divided into the GLP-1 RAs group (3344 patients) and the control group (2891 patients). The primary endpoint was to assess the degree of bowel preparation using the Boston Bowel Preparation Scale (BBPS). The data indicated that patients taking GLP-1 RAs had a lower quality of bowel preparation than controls; in particular, in the group on GLP-1 RAs therapy, the total BBPS score was significantly lower. These patients were more likely to have inadequate preparation (26.4% vs. 18.7% in controls). Interestingly, the adenoma detection rate (ADR) was higher in the GLP-1 RAs group (48.8% vs. 42.3%), probably due to comorbidities (e.g., obesity).

## 6. Therapeutic Potential of GLP-1 Modulation in IBD

### 6.1. What Are Preclinical Potentials and Underlying Mechanisms?

**Figure 2:**
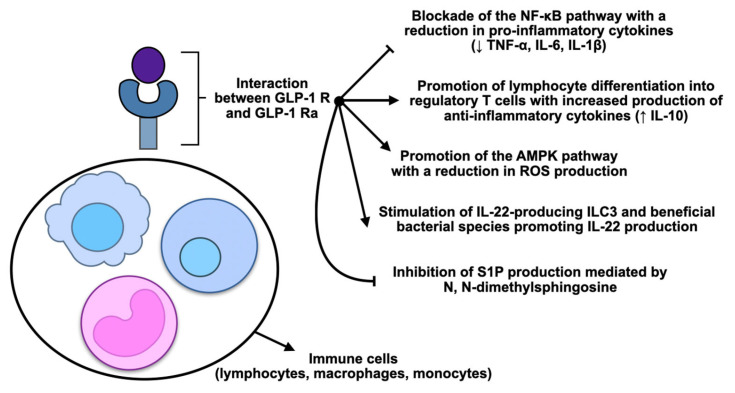
Main molecular mechanisms postulated for an anti-inflammatory immunological action of glucagon-like peptide-1 receptor (GLP-1 R) modulation by receptor agonists (GLP-1 Ra) at the level of immune cells (primarily lymphocytes, macrophages, and monocytes). Acronyms: NF-κB: nuclear factor kappa-light-chain-enhancer of activated B cells; TNF-α: tumour necrosis factor-alpha; IL: interleukin; AMPK: AMP-activated protein kinase; ROS: reactive oxygen species; ILC3: group 3 innate lymphoid cells; S1P: sphingosine-1-phosphate.

For the reasons outlined above, GLP-1 modulators could, therefore, be de jure considered of interest to explore for their potential therapeutic application in IBD. Preclinical trials and initial clinical data indicate that its receptor activation might promote glycaemic modulation and the downregulation of intestinal inflammatory events [86]. Specifically, the action of GLP-1 seems to diminish proinflammatory cytokine production and induce epithelial barrier restitution and immune balance. These effects, combined with improved metabolic function, offer a dual benefit that could effectively complement currently available therapies for IBD. Given the already mentioned short half-life due to rapid enzymatic degradation [19], DPP-4-resistant GLP-1 analogues, including exenatide, liraglutide, albiglutide, dulaglutide, which are FDA-approved and characterised by an increased half-life, have been developed [86]. These GLP-1 RAs modulate inflammatory pathways and influence several chronic diseases, from diabetes to cardiovascular diseases, including neurodegenerative diseases and IBD [87]. It has been observed that GLP-1 RAs may have anti-inflammatory properties in IBD by regulating immune cell signalling [55]. Reduced production of proinflammatory cytokines and increased activity of regulatory T-cells are some of the mechanisms through which GLP-1 RAs protect against intestinal inflammation in models of early-stage IBD [88]. These receptor agonists act by interacting with GLP-1 receptors present in immune cells such as lymphocytes, monocytes and macrophages. Their binding results in a modulation of intracellular signalling pathways, reducing the production of proinflammatory cytokines (e.g., TNF-α, IL-6, and IL-1β) and an increase in anti-inflammatory cytokines (i.e., IL-10). GLP-1 RAs also act by inhibiting the activation of the nuclear factor κ-light-chain-enhancer of activated B cells (NF-κB) pathway and stimulating the AMP-activated protein kinase (AMPK) signalling pathway, thus helping to limit the inflammatory response and reducing the production of reactive oxygen species (ROS). GLP-1 RAs protect cells from inflammatory damage by reducing oxidative stress and promoting tissue homeostasis through these mechanisms. GLP-1RAs influence gut microbiota profiling and gut barrier status, both of which alterations contribute to the patient’s IBD pathogenesis [89]. In a 2023 study, Wang et al. [90] demonstrated that intraperitoneal injection of GLP-1 reduces the effect of tissue damage in mice dextran sodium sulphate (DSS)-induced colitis by inhibiting intestinal inflammation, maintaining the gut barrier and regulating the gut microbiota. Indeed, GLP-1 exerts a protective effect in DSS-induced colitis mice by regulating key signalling pathway participants: Ak strain transforming (AKT)/NF-κB p65 and mitogen-activated protein kinase (MAPK). In vitro, GLP-1 was also shown to inhibit inflammatory pathways in lipopolysaccharide (LPS)-induced Received Abelson leukaemia virus-transformed Weighty 264.7 (RAW264.7) macrophage lineage cells, as the level of phosphorylated NF-κB-p65 and AKT was higher in these cells. Their levels were restored with GLP-1 treatment, similar to what was shown in the mouse model. There was also a significant reduction in the expression of zonulin-1 and occludins in the DSS-treated group, while GLP-1 treatment restored them, suggesting that GLP-1 may prevent the breakdown of the mucosal barrier by inhibiting the abnormal immune response in the intestinal mucosa. Intestinal barrier impairment and mucosal inflammation are frequently accompanied by a reduction in beneficial bacterial species in the gut [1]. In this study, GLP-1 led to an increase in the richness of the microbiota, in particular favouring the survival and proliferation of the *Lactobacillaceae* and *Bifidobacteriaceae* families, which are known for their role in modulating immune responses [91]. It has also been shown that GLP-1 RAs not only act by modulating the cytokines involved in IBD but also act on innate lymphoid type 3 cells (ILC3), which are involved in defence against pathogens and repair of the intestinal mucosa. In mice with DSS-induced colitis, administration of liraglutide slowed weight loss and preserved colonic length to a greater extent and improved the disease activity index (DAI) due to increased ILC3-producing IL-22. The loss of benefit shown in mice lacking IL-22-producing ILC3s, together with the efficacy maintained in animals lacking T- and B-lymphocytes, confirms that ILC3s are essential for the anti-inflammatory action of the drug. At the same time, the therapy modified the microbiota by making it more similar to that of healthy subjects, with an increase in bacteria such as *Firmicutes* and specific microbes such as *Lactobacillus reuteri*, *Lactobacillus johnsonii* and *Helicobacter typhlonius*, known to stimulate IL-22 production [92,93,94,95]. Finally, an increase in N, N dimethylsphingosine (DMS) levels was observed, which inhibits the formation of sphingosine 1 phosphate (S1P), a proinflammatory molecule involved in lymphocyte recruitment in tissues. S1P exerts its proinflammatory effects primarily by binding to the S1PR1 receptor on lymphocytes, thereby promoting their migration into inflamed tissues [96,97]. High S1P concentrations have been observed in the intestinal mucosa of patients with IBD. S1PR modulators such as fingolimod, ozanimod, and etrasimod have emerged as novel small-molecule drugs for the treatment of IBD [96,97]. The liraglutide-induced increase in DMS levels strengthens the epithelial barrier and reduces intestinal inflammation. These results indicate that GLP-1 RAs may promote mucosal healing and modulate the microbiota–metabolite axis to counteract colitis, offering new insights for targeted therapies in IBD [98]. Figure 2 summarises the principal anti-inflammatory mechanisms mediated by GLP-1 modulation through GLP-1 RAs.

### 6.2. What Data Are Already Available in Human Contexts, and What Are the Possible Considerations?

Preclinical animal studies have provided such encouraging results that human clinical trials have been initiated, with promising outcomes in both short bowel syndrome (SBS) and chronic IBD. In the most severe cases of IBD, patients may require surgical resections that can lead to SBS and its complications [99,100]. Moreover, in patients with CD, there is also a certain risk of chronic intestinal failure, which has been estimated at approximately 1%, according to a recent Italian multicentre study [101]. In another recent Canadian study on nearly four hundred patients with intestinal failure, SBS related to CD accounted for approximately 45% of cases [102].

Following the approval of exendin-4 (a GLP-1 receptor agonist) for T2DM, a small retrospective analysis of five patients with SBS was conducted: exendin-4 reduced antral contractions both on an empty stomach and after meals, slowed gastric emptying and prolonged chyme contact with the absorbing mucosa, resulting in improved evacuation frequency and reduced need for total parenteral nutrition [103]. In another open-label, placebo-controlled study of nine patients with SBS, GLP-1 infusion reduced diarrhoea and evacuation frequency, albeit to a lesser extent than GLP-2. Teduglutide, a DPP4-resistant, long half-life GLP-2 analogue, has already been approved for SBS and is being investigated in IBD [104,105]. However, co-infusion of GLP-1 and GLP-2 has an additive effect on intestinal absorption compared to either peptide alone [104]. In addition, co-administration of GLP-1 and GLP-2 can reduce the side effects commonly seen with GLP-1 alone, such as nausea and loss of appetite. Since the impacts on the intestinal system deserve special attention, any GLP-1-based therapy should be started gradually, starting with small dosages and choosing short- or long-term therapy—best suited to individual needs. Looking ahead, developing molecules with dual GLP-1/GLP-2 activity or including GLP-1 agonists in multimodal therapy schemes could help minimise these disorders [106]. In one of the first studies in which GLP-1 was tested on IBD, it was shown that the human GLP-1 (7-36) self-associated with PEGylated phospholipid micelles (GLP-1-SSM) partially reduced the diarrhoeal phenotype in mice with DSS colitis through two potential mechanisms: reduction in colonic inflammation and preservation of DRA (down-regulated in adenoma) expression. DRA is the main Cl^−^/HCO_3_^−^ exchanger in the gut. Several studies have shown repression of DRA expression in the inflamed gut, and it has been identified as a novel risk factor for the development of UC [107].

A case report exploring GLP-1 RA treatment, specifically liraglutide, in a patient with UC was published in 2019 [108]. In this case, administration of daily subcutaneous injections of liraglutide (0.6 mg titrated to 3.0 mg) for the treatment of obesity resulted in complete remission of colitis symptoms with minimal adverse effects, limited to mild nausea associated with dose titration. In an Israeli national cohort of patients with IBD (epi-IIRN), data were retrieved from 3737 patients with IBD and T2DM, of whom 633 were treated with GLP-1 RAs for a median follow-up of 6 years per patient. The primary endpoint was poor IBD-related outcomes defined as a composite of events such as steroid dependence, IBD treatment escalation, IBD-related hospitalisation, abdominal/perianal surgery or death. GLP-1 RAs are associated with a reduced risk of adverse disease outcomes. A sub-analysis also shows that using GLP-1 analogues is associated with an improved IBD disease course, specifically in patients with obesity [109]. However, these GLP-1 analogues and high production costs may have reduced bioactivity and an increased risk of immunogenicity.

Therefore, a nanocarrier system with sterically SSM, to which native human GLP-1 self-associates, was designed. This carrier system increases the in vivo stability of GLP-1 while maintaining its anti-inflammatory properties. Furthermore, due to the carrier’s nanometric size (~14 nm), the SSM-associated peptide can only escape from circulation into the permeable vessels of inflamed tissues via the resulting enhanced permeability and retention effect with passive targeting. This targeted effect should increase the peptide’s activity for a given dose and eliminate the peptide’s toxicity for healthy tissues [110]. As previously reported, circulating levels of GLP-1 are primarily dependent on the activity of endopeptidases such as CD26/DPP-4. Increased expression of CD26 on T-cells is observed in IBD patients despite a decrease in systemic activity [111,112]. Since modifications of CD26 are involved in many immune-mediated diseases—from autoimmune diabetes to graft-versus-host disease and to anti-tumour response and IBD—this enzyme has become a therapeutic target [113]. CD26 inhibitors such as sitagliptin, linagliptin, vildagliptin, alogliptin, saxagliptin, teneligliptin and anagliptin have been shown, in preclinical models and clinical trials, to be safe and effective in modulating pathological immune responses [114,115,116]. Recent studies have suggested that administering CD26 inhibitors may reduce inflammation in the colon [117,118,119]. Another mechanism of GLP-1 modulation involves the G-protein-coupled receptor 120 (GPR120) of the rhodopsin family, which is highly present in the colon and sensitive to dietary fat composition and inflammatory status [120,121]. Activation of GPR120 promotes the release of incretins, including GLP-1, and is also found on CD4^+^ T cells, key players in the pathogenesis of IBD [122,123]. The expression of GPR120 and the availability of GLP-1 in the mucosa also depend on the balance of the microbiota—in particular of the phyla *Firmicutes*, *Bacteroides* and *Proteobacteria*—which is altered in subjects with IBD [124]. Activation of this receptor, which is also expressed in immune cells such as macrophages and T lymphocytes, has been shown to suppress the production of proinflammatory cytokines, reducing disease severity in mouse models of colitis [121,125].

## 7. Conclusions

In conclusion, modulation of the GLP-1 system could represent a novel therapeutic approach for treating IBD. However, well-structured clinical studies are crucial to elucidate the mechanisms of their anti-inflammatory effects and to confirm both the safety and efficacy of these agents, helping to guide future therapeutic choices. Several clinical trials are currently being conducted to further investigate the role of GLP-1 modulation in the field of IBDs, as shown in Table 1.

Taking into account all the considerations expressed so far, the following can be regarded as fixed points in approaching this subject:

- GLP-1 RAs have demonstrated efficacy in controlling body weight and improving glycaemic parameters in patients with IBD, with a performance comparable to that observed in the general population.
- GLP-1 RAs appear to possess various anti-inflammatory properties and potentials within the gastrointestinal tract, modulating the mucosal immune response and positively influencing epithelial barrier homeostasis. This is further supported by the expression of GLP-1 receptors in segments highly affected by IBD, such as the terminal ileum and the rectum.
- Initial and preliminary evidence seems to suggest that patients with IBD treated with GLP-1 RAs may experience a benefit in terms of reduced surgery rates and hospitalisations, although further studies are warranted.
- Although GLP-1 RAs are overall drugs with an acceptable safety profile, they do present a non-negligible risk of gastrointestinal adverse events and a potential impact on bowel preparation for endoscopic procedures, aspects that must be considered when used in patients with IBD.
- In the future, large-scale prospective randomised clinical trials are therefore needed to assess the efficacy and safety of GLP-1 RAs in terms of clinically, endoscopically, and histologically relevant outcomes specific to IBD. It is necessary to understand the long-term effects of IBD on the natural history of GLP-1 receptor modulation.

## Figures and Tables

**Table 1:** List of ongoing studies on the modulation of glucagon-like peptide-1 (GLP-1) and its receptor in inflammatory bowel disease (IBD).

ID ^1^	Title	Design (Phase)	Arm(s)	Primary Outcomes
NCT06774079	A Randomized Clinical Trial to Determine the Effect of Dual Glucose-dependent Insulinotropic Polypeptide (GIP)/GLP-1 Receptor Agonist-mediated Weight Loss and Diet on Crohn’s Disease Clinical Response: a Pilot Study	RCT (Phase 4)	(1) Tirzepatide and Mediterranean diet versus (2) Mediterranean diet	Change in percentage of participants retained; number of patients who adhere to diet measured by 24 h dietary recall; number of patients who adhere to medications measured by self-report
NCT05196958	Interest of GLP1 Analogues (aGLP1) in Overweight Type 2 Diabetic Patients With Chronic Inflammatory Bowel Disease (IBD) (DiagMICI)	nRCT (N/A)	(1) GLP-1 analogues	Glycaemic control; weight loss

^1^ The study ID is derived from the ClinicalTrials.gov platform (https://clinicaltrials.gov/), accessed on 15 May 2025). Acronym: RCT: randomised controlled trial; nRCT: nonrandomised controlled trial; N/A: not applicable.
